# Poly(tris(4-carbazoyl-9-ylphenyl)amine)/Three Poly(3,4-ethylenedioxythiophene) Derivatives in Complementary High-Contrast Electrochromic Devices

**DOI:** 10.3390/polym9100543

**Published:** 2017-10-23

**Authors:** Chung-Wen Kuo, Jeng-Kuei Chang, Yuan-Chung Lin, Tzi-Yi Wu, Po-Ying Lee, Tsung-Han Ho

**Affiliations:** 1 Department of Chemical and Materials Engineering, National Kaohsiung University of Applied Sciences, Kaohsiung 80778, Taiwan; welly@cc.kuas.edu.tw (C.-W.K.); k40017105@gcloud.csu.edu.tw (P.-Y.L.); thho@cc.kuas.edu.tw (T.-H.H.); 2 Institute of Materials Science and Engineering, National Central University, Taoyuan 32001, Taiwan; jkchang@ncu.edu.tw; 3 Institute of Environmental Engineering, National Sun Yat-Sen University, Kaohsiung 80424, Taiwan; yclin@faculty.nsysu.edu.tw; 4 Department of Chemical Engineering and Materials Engineering, National Yunlin University of Science and Technology, Yunlin 64002, Taiwan

**Keywords:** electrochemical polymerization, optical contrast, electrochromic switching, coloration efficiency, electrochromic device

## Abstract

A carbazole-based polymer (poly(tris(4-carbazoyl-9-ylphenyl)amine) (PtCz)) is electrosynthesized on an indium tin oxide (ITO) electrode. PtCz film displays light yellow at 0.0 V, earthy yellow at 1.3 V, grey at 1.5 V, and dark grey at 1.8 V in 0.2 M LiClO_4_/ACN/DCM (ACN/DCM = 1:3, by volume) solution. The Δ*T* and coloration efficiency (*η*) of PtCz film are 30.5% and 54.8 cm^2^∙C^−1^, respectively, in a solution state. Three dual-type electrochromic devices (ECDs) are fabricated using the PtCz as the anodic layer, poly(3,4-ethylenedioxythiophene) (PEDOT), poly(3,3-dimethyl-3,4-dihydro-thieno[3,4-*b*][1,4]dioxepine) (PProDOT-Me_2_), and poly(3,4-(2,2-diethylpropylenedioxy)thiophene) (PProDOT-Et_2_) as the cathodic layers. PtCz/PProDOT-Me_2_ ECD shows high Δ*T*_max_ (36%), high *η*_max_ (343.4 cm^2^·C^−1^), and fast switching speed (0.2 s) at 572 nm. In addition, PtCz/PEDOT, PtCz/PProDOT-Me_2_, and PtCz/PProDOT-Et_2_ ECDs show satisfactory open circuit memory and long-term stability.

## 1. Introduction

π-conjugated polymers (CPs) and oligomers have attracted a great deal of interest due to their suitability for potential applications in supercapacitors [1,2], catalysts [3,4,5], actuators [6], polymer light-emitting diodes [7,8,9], electrochromic devices (ECDs) [10,11,12], polymer solar cells [13], and sensors [14,15,16]. The most commonly studied classes of CPs are poly(phenylene vinylene)s (PPV) [17], polycarbazoles (PCz) [18,19], polythiophenes (PT) [20], polypyrroles (PPy) [21], poly(3,4-ethylenedioxythiophene) (PEDOT) [22], and polyanilines (PANI) [23]. Cz-based polymers have been widely used as hole transporting and host materials in optoelectronic devices due to the nitrogen atom of Cz ring shows good hole transporting ability, high thermal stability, and ease of formation of radical cations and dications [24]. Polythiophenes and polypyrroles have been extensively used as electrochromic materials due to the fact that they can be easily synthesized electrochemically or chemically with a wide range of electrochromic properties available through alkyl, alkoxy, and phenyl substitution on polythiophenes and polypyrroles. PEDOT and its derivatives poly(3,3-dimethyl-3,4-dihydro-thieno[3,4-*b*][1,4]dioxepine) (PProDOT-Me_2_) and poly(3,3-diethyl-3,4-dihydro-thieno[3,4-*b*][1,4]dioxepine) (PProDOT-Et_2_) were extensively investigated for many useful properties including low oxidation potential, electron-rich dioxy group, optical transparency in doped state, moderate band gap, and high stability [25,26].

There have been no reports for the applications of poly(tris(4-carbazoyl-9-ylphenyl)amine) as anodic polymer in electrochromic devices. In the present study, a carbazole-based monomer (tris(4-carbazoyl-9-ylphenyl)amine, tCz) was synthesized and its corresponding homopolymer (PtCz) was polymerized electrochemically. The spectroelectrochemistry, electrochromic photographs, optical contrast, and coloration efficiency of PtCz film in solution state were studied. Moreover, dual-type ECDs based on PtCz and PEDOT derivatives were fabricated, the electrochromic behaviors, open circuit memory, and long-term switching stability of PtCz/PEDOT, PtCz/PProDOT-Me_2_, and PtCz/PProDOT-Et_2_ ECDs were also investigated.

## 2. Materials and Methods

### 2.1. Materials

All chemicals were purchased from Sigma-Aldrich, Tokyo Chemical Industry Co., Ltd. (TCI, Tokyo, Japan), Acros (Geel, Belgium), Alfa-Aesar (Ward Hill, MA, USA), and used as received. Tris(4-carbazoyl-9-ylphenyl)amine, 3,3-dimethyl-3,4-dihydro-thieno[3,4-*b*][1,4]dioxepine (ProDOT-Me_2_) and 3,3-diethyl-3,4-dihydro-thieno[3,4-*b*][1,4]dioxepine (ProDOT-Et_2_) were synthesized following previously published procedures [27,28].

### 2.2. Synthesis of Tris(4-carbazoyl-9-ylphenyl)amine (tCz)

Carbazole (70.22 mg, 0.42 mmol), tris(4-iodophenyl)amine (68.53 mg, 0.11 mmol), K_2_CO_3_ (165.84 mg, 1.20 mmol), Cu bronze (69.58 mg, 1.095 mmol) and 18-crown-6 (8.72 mg, 0.033 mmol) were stirred in 45 mL 1,2-dichlorobenzene for two days at 190 °C. The crude product is vacuum distilled and the residue is purified by column chromatography using a mixture of hexane and dichloromethane (DCM) (2:1 by volume) as eluent. Yield: 41%. ^1^H-NMR (700 MHz, DMSO-*d*_6_): δ 8.26 (d, 6H, Ar-H), 7.69 (dd, 6H, Ar-H), 7.57 (dd, 6H, Ar-H), 7.50–7.46 (m, 12H, Ar-H), 7.32–7.30 (m, 6H, Ar-H). Elem. anal. calcd. for C_54_H_36_N_4_: C, 87.54%; H, 4.90%; N, 7.56%. Found: C, 87.32%; H, 4.82%; N, 7.57%.

### 2.3. Electrosynthesis of PtCz, PProDOT-Me_2_, and PProDOT-Et_2_ Films

The electrosynthesis of PtCz film in an ACN/DCM (1:3, by volume) solution containing 0.2 M LiClO_4_ as a supporting electrolyte was carried out by scanning the potential between 0.0 and 1.8 V (vs. Ag/AgCl) potentiodynamically at 100 mV·s^−1^ for 3 cycles. The electrochemically deposited PtCz film was rinsed with DI water for 5 min and then dried at 105 °C for 3 min. As shown in Table 1, the PEDOT, PProDOT-Me_2_, and PProDOT-Et_2_ films were deposited from 0.008 M EDOT, 0.010 M ProDOT-Me_2_, and 0.017 M ProDOT-Et_2_ in a 0.2 M LiClO_4_/acetonitrile (ACN) solution, respectively. Electrosynthesis of PEDOT, PProDOT-Me_2_, and PProDOT-Et_2_ films were performed potentiostatically at 1.7 V (vs. Ag/AgCl) for 50 mC. Polymer thicknesses at the electrode surface obtained from an Alpha-Step profilometer (KLA Tencor D-120, CA, USA) were about 200–300 nm.

### 2.4. Electrochromic Characterization

Electrochromic characterization of the polymer films and electrochromic devices were carried out using a CHI627D electrochemical analyzer (CH Instruments, Austin, TX, USA). Cyclic voltammetry (CV) studies were performed using in a three-component cell, which contained an ITO-coated glass plate (area: 1 cm × 1.5 cm) as the working electrode, a platinum wire as the counter electrode, and an Ag/AgCl as the reference electrode. The in situ spectroelectrochemical spectra were recorded using an Agilent Cary 60 UV-Visible spectrophotometer (Varian Inc., Walnut Creek, CA, USA) in time course mode.

### 2.5. Preparation of Electrochromic Electrolytes

The polymer electrolytes of the ECDs were prepared using the solution-cast method. To prepare the solution, poly(methyl methacrylate) (PMMA), propylene carbonate (PC), and LiClO_4_ were dissolved in acetone, and the mixture was stirred magnetically at room temperature for 36 h. The polymer electrolytes were prepared using PMMA:PC:LiClO_4_ in a weight ratio of 33:53:14. The final mixture was cast on glass petri dishes. After evaporating the solvent at room temperature for 2 h, the samples were vacuum-dried at 80 °C for 24 h to remove the remaining solvent completely. Finally, the self-standing polymer electrolytes were obtained. The ECDs were fabricated by sandwiching the polymer electrolytes between two electrodes to perform the electrochromic measurements.

### 2.6. Fabrication of the ECDs

**Figure 1:**
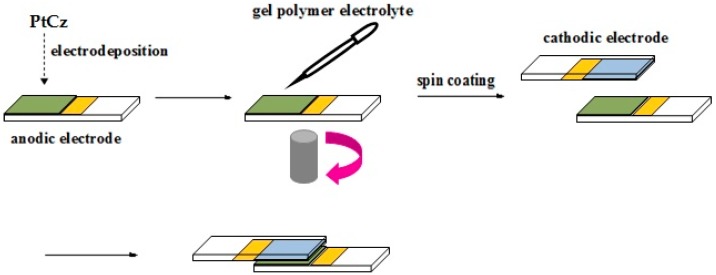
The fabrication procedures of electrochromic devices (ECDs).

The ECDs were constructed using two complementary polymer layers, PtCz as the anodically coloring layer, PEDOT, PProDOT-Me_2_, or PProDOT-Et_2_ as the cathodically coloring layer. PtCz, PEDOT, PProDOT-Me_2_, and PProDOT-Et_2_ films were deposited on ITO substrates (active area: 1 cm × 1.5 cm). The ECDs were fabricated by arranging the oxidized and reduced films to face each other, and they were separated by an electrolyte. The fabrication procedures of ECDs are shown in Figure 1.

## 3. Results and Discussion

### 3.1. Electrochemistry of tCz and Its Electrochemical Polymerization

**Figure 2:**
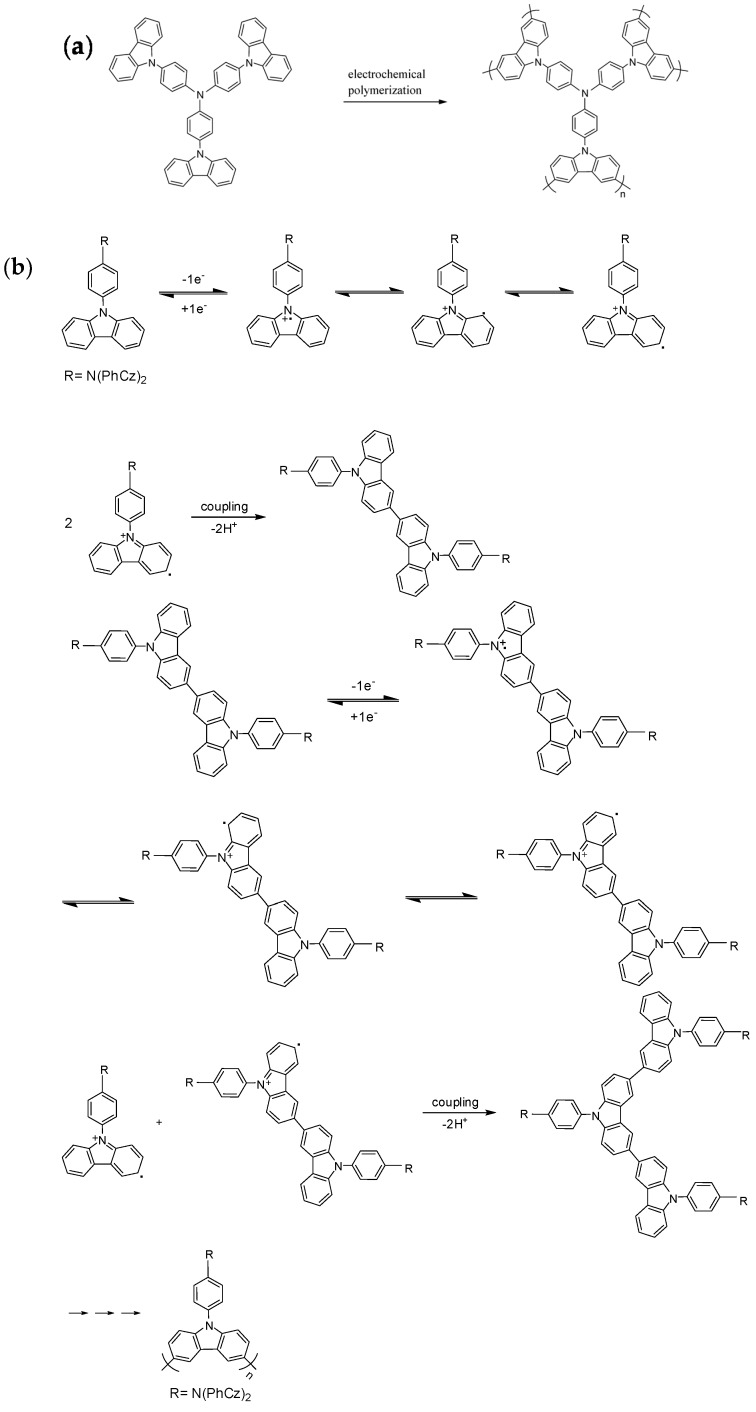
(**a**) The electrochemical polymerization scheme of the carbazole-based polymer (poly(tris(4-carbazoyl-9-ylphenyl)amine) (PtCz)); (**b**) the electropolymerization mechanism of PtCz.

**Figure 3:**
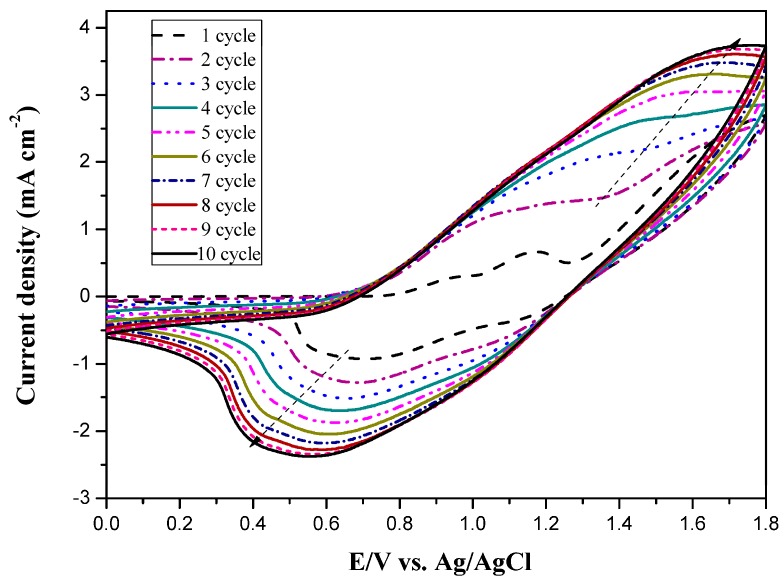
Electrochemical synthesis of PtCz in acetonitrile (ACN)/dichloromethane (DCM) (1:3, by volume) solution at 100 mV∙s^−1^ on indium tin oxide (ITO) working electrode.

The electrosynthesis of PtCz film was implemented using CV with a scan rate of 100 mV∙s^−1^. The electropolymerization scheme and mechanism of PtCz are shown in Figure 2 [29]. The successive cyclic voltammograms of 0.002 M neat tCz taken in an ACN/DCM (1:3, by volume) solution containing 0.2 M LiClO_4_ as a supporting electrolyte at a scanning rate of 100 mV·s^−1^ are shown in Figure 3. For the first scan of cyclic voltammogram, the onset potential of tCz is 0.86 V vs. Ag/AgCl, two oxidation peaks located at 0.95 and 1.18 V indicate the polaron and bipolaron formation of tCz, the reduction peaks of tCz locate at 1.1 and 0.7 V. The increase in the oxidation and reduction curves wave current densities indicates that the amount of polymer deposited on the ITO working electrode increases with increasing cycles. The polymerization of tCz shows two quasi-reversible oxidation and reduction processes in Figure 3.

### 3.2. Electrochemical Behavior of PtCz Films

**Figure 4:**
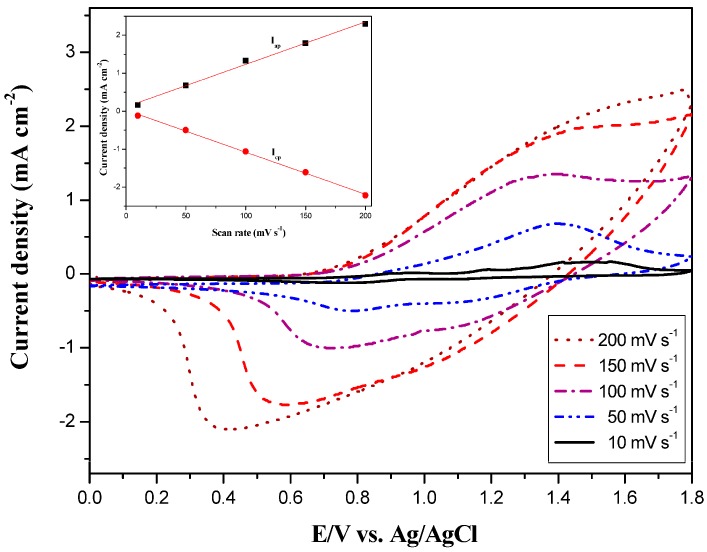
Cyclic voltammetry (CV) curves of the PtCz film-coated ITO working electrode at different scan rates between 10 and 200 mV∙s^−1^ in 0.2 M LiClO_4_/ACN/DCM solution. Inset: Scan rate dependence of the PtCz anodic and cathodic peak current densities, respectively.

The as-prepared PtCz film was swept between 0.0 to 1.8 V at various scan rates between 10 and 200 mV·s^−^^1^ in 0.2 M LiClO_4_/ACN/DCM solution. As shown in Figure 4, the electrochemical behavior of the PtCz film shows a single well-defined redox process, the anodic and cathodic peak current densities are proportional to the scan rates, implying that PtCz film is electroactive and adheres well to the electrode, and the electrochemical processes of PtCz film are reversible and not dominated by diffusion effects [30].

### 3.3. Spectroelectrochemistry of PtCz and PProDOT-Me_2_ Films

**Figure 5:**
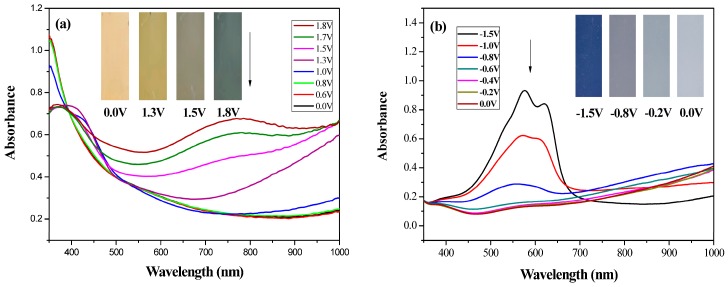
UV-Visible spectra of (**a**) PtCz and (**b**) PProDOT-Me_2_ on ITO in an ACN/DCM (1:3, by volume) solution containing 0.2 M LiClO_4_.

Spectroelectrochemistry can be used to analyze the changes in the absorption spectra of ECDs at various potentials [31]. Optoelectrochemical spectra of PtCz and PProDOT-Me_2_ films are shown in Figure 5. The PtCz film shows a π-π* transition peak at around 360 nm at 0.0 V, and it is light yellow in undoped state. Upon stepwise oxidation, the peak intensity at 360 nm diminishes gradually and new absorption bands at around 800 nm emerge, the PtCz film displays earthy yellow at 1.3 V, grey at 1.5 V, and dark grey at 1.8 V. On the other hand, the PProDOT-Me_2_ film shows two significant peaks at 570 and 625 nm at −1.5 V and presents dark blue in its neutral state. Upon oxidation progressively, the peak intensity at 570 and 625 nm diminish gradually and new absorption bands at more than 1000 nm emerge, the PProDOT-Me_2_ film displays grey at −0.8 V and light blue at −1.5 V. The colorimetric values (L*, a*, and b*), CIE chromaticity values (*x*, *y*), and CIE chromaticity diagrams of the PtCz and PProDOT-Me_2_ films at various potentials were shown in Table 2.

### 3.4. Electrochemical Switching of PtCz Film

**Figure 6:**
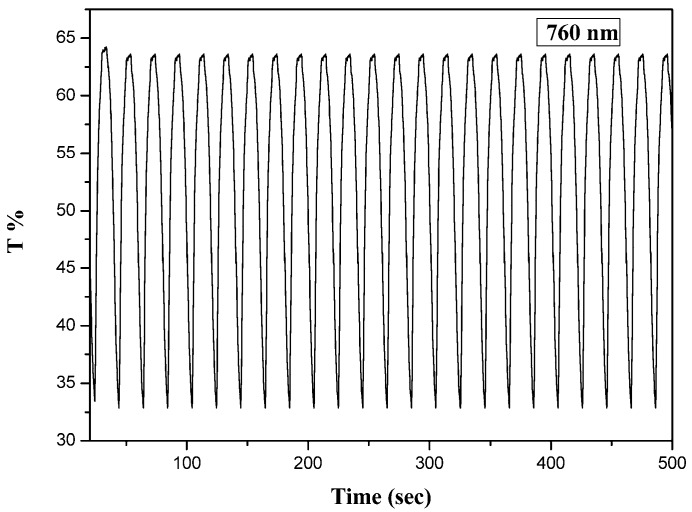
Optical contrast of PtCz electrode in an ACN/DCM (1:3, by volume) solution containing 0.2 M LiClO_4_ between 0.0 V and 1.8 V with a residence time of 10 s.

Double potential step techniques can be used to investigate the response time and stability of polymer films during consecutive scans [32]. The double potential step chronoamperometry coupled with spectrophotometer of PtCz film was performed by stepping potentials between 0.0 and 1.8 V with a residence time of 10 s, and the transmittance-time profile of PtCz film is displayed in Figure 6. The coloration switching time (*τ*_c_) and bleaching switching time (*τ*_b_) were defined as the period required for achieving 90% of the desired response [33,34,35,36]. The *τ*_c_ and *τ*_b_ of PtCz film estimated at the third cycle at 760 nm are 5.5 and 5.0 s, respectively. The optical contrast (Δ*T*%) is an important property of electrochromic polymer films, which denotes as the transmittance difference between bleaching and coloring states of polymer films in solution state. The optical density (ΔOD) can be calculated using the following formula:

(1) $$\Delta \mathrm{OD}=\log(\frac{T_{\mathrm{ox}}}{T_{\mathrm{red}}})$$

where *T*_ox_ is the transmittance of anodic material in coloration state and *T*_red_ is the transmittance of anodic material in bleaching state.

As shown in Table 3, the Δ*T*_max_ and ΔOD values of PtCz film are 30.5% and −0.28, respectively, at 760 nm in 0.2 M LiClO_4_/ACN/DCM (ACN/DCM = 1:3, by volume) solution. The Δ*T*_max_ of PtCz film is larger than those reported for poly(9*H*-carbazol-9-ylpyrene) (Δ*T*_max_ = 29% at 460 nm [37]) and poly(1,3-bis(carbazol-9-yl)benzene) (Δ*T*_max_ = 18.6% at 1050 nm [38]) (Table 4). However, the Δ*T* of PtCz film is smaller than those reported for poly(ethyl-4-(3,6-di(thiophen-2-yl)-9*H*-carbazole-9-yl)-benzoate) (Δ*T*_max_ = 36% at 1100 nm [39]), poly(2,5-bis(9-methyl-9*H*-carbazol-3-yl)-1,3,4-oxadiazole) (Δ*T*_max_ = 75% at 660 nm [40]), poly(3,6-di(carbazol-9-yl)-*N*-(4-nitrophenyl)carbazole) (Δ*T*_max_ = 52% at 710 nm [29]), and poly(4,4′-bis(*N*-carbazolyl)-1,1′-biphenyl) (Δ*T*_max_ = 44.1% at 800 nm) [41].

The coloration efficiency (*η*) of electrochromic materials can be estimated using the following equation [42]:

(2) $$\eta =\frac{\Delta \mathrm{OD}}{Q_{d}}$$

where *Q*_d_ is the charge density (injected/ejected charges per unit sample area). The *η* value of PtCz film is 54.8 cm^2^∙C^−1^ at 760 nm in 0.2 M LiClO_4_/ACN/DCM (ACN/DCM = 1:3, by volume) solution. The *η* of PtCz film is larger than that reported for poly(3,6-di(carbazol-9-yl)-*N*-(4-nitrophenyl)carbazole) (*η* = 35 cm^2^∙C^−1^ [29]). However, the Δ*T*_max_ of PtCz film is smaller than those reported for poly(4,4′-bis(*N*-carbazolyl)-1,1′-biphenyl (*η* = 98 cm^2^∙C^−1^ [41]) and poly(1,3-bis(carbazol-9-yl)benzene) (*η* = 180.3 cm^2^∙C^−1^ [38]).

### 3.5. Spectroelectrochemistry of PtCz/PEDOT, PtCz/PProDOT-Me_2_, and PtCz/PProDOT-Et_2_ ECDs

**Figure 7:**
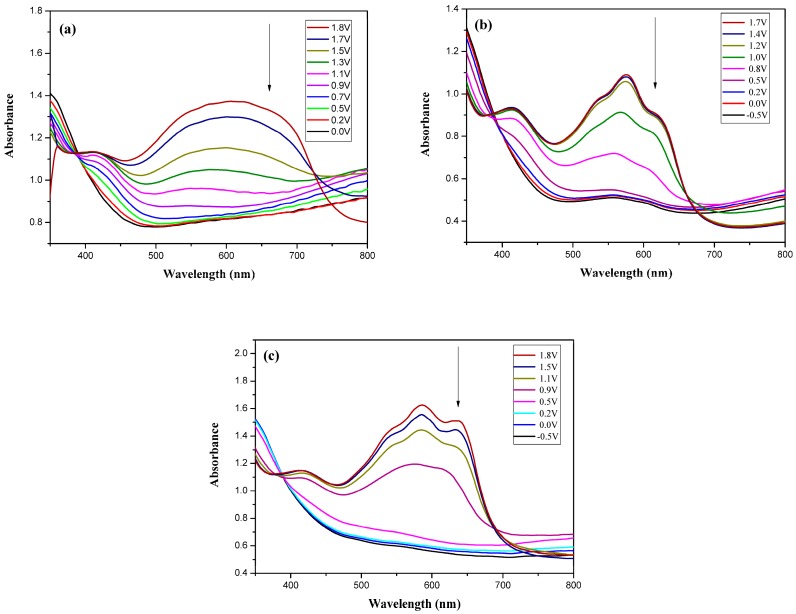
UV-visible spectra of (**a**) PtCz/PEDOT (**b**) PtCz/PProDOT-Me_2_, and (**c**) PtCz/PProDOT-Et_2_ ECDs.

Figure 7 shows the UV-Visible spectra of PtCz/PEDOT, PtCz/PProDOT-Me_2_, and PtCz/PProDOT-Et_2_ ECDs at various voltages. At 0.0 V, the PtCz/PEDOT, PtCz/PProDOT-Me_2_, and PtCz/PProDOT-Et_2_ ECDs show π-π* transition peaks of PtCz film at around 360 nm. 

Upon increasing the potential gradually, the π-π* transition peak of PtCz film diminishes and new absorption band at around 580–650 nm emerges. At 1.7–1.8 V, PEDOT, PProDOT-Me_2_, and PProDOT-Et_2_ films exhibit distinct absorption band at around 500–700 nm, and PtCz/PEDOT, PtCz/PProDOT-Me_2_, and PtCz/PProDOT-Et_2_ ECDs became dark blue at 1.7–1.8 V. The electrochromic photographs, colorimetric values (L*, a*, and b*), CIE chromaticity values (*x*, *y*), and CIE chromaticity diagram of the PtCz/PProDOT-Me_2_ ECD at various potentials are summarized in Table 5.

**Figure 8:**
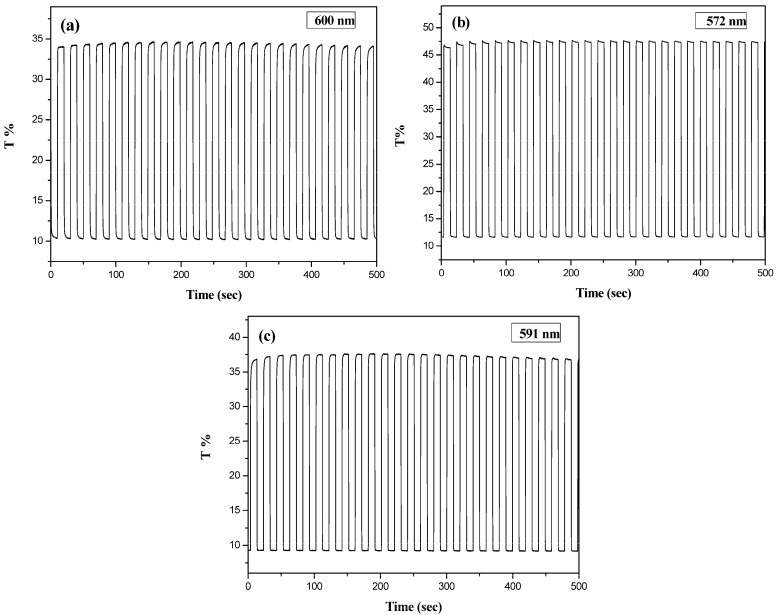
Optical contrast of (**a**) PtCz/PEDOT (**b**) PtCz/PProDOT-Me_2_, and (**c**) PtCz/PProDOT-Et_2_ ECDs in an ACN/DCM (1:3, by volume) solution containing 0.2 M LiClO_4_ between 0.0 V and 1.8 V with a residence time of 10 s.

Figure 8 shows the transmittance-time profiles of PtCz/PEDOT, PtCz/PProDOT-Me_2_, and PtCz/PProDOT-Et_2_ ECDs by repeating potentials between 0.0 and 1.8 V with a time interval of 10 s. The *τ*_c_ and *τ*_b_ estimated at various cycles for PtCz/PEDOT, PtCz/PProDOT-Me_2_, and PtCz/PProDOT-Et_2_ ECDs are listed in Table 3. The *τ*_c_ and *τ*_b_ of PtCz/PEDOT ECD at 600 nm were 0.2 and 0.2 s, respectively, at the 50th cycle. Under similar conditions, the *τ*_c_ values of PtCz/PProDOT-Me_2_ ECD at 572 nm and PtCz/PProDOT-Et_2_ ECD at 591 nm were 0.2 and 0.1 s at the 50th cycle, respectively, and the corresponding *τ*_b_ values were 0.2 and 0.1 s at the 50th cycle, respectively, indicating that PtCz/PEDOT, PtCz/PProDOT-Me_2_, and PtCz/PProDOT-Et_2_ ECDs showed fast switching speed when PtCz, PEDOT, PProDOT-Me_2_, and PProDOT-Et_2_ are employed as electrochromic layers. The *τ*_c_ and *τ*_b_ values of PtCz/PEDOT ECD were 0.6 and 1.0 s, respectively, at the third cycle, and 0.2 and 0.2 s, respectively, at the 50th cycle, indicating that switching time shortened with the number of switching cycles. Under similar conditions, PtCz/PProDOT-Me_2_ and PtCz/PProDOT-Et_2_ ECDs showed fast switching speed at high switching cycles than those at low switching cycles.

As summarized in Table 3, the Δ*T* values of PtCz/PEDOT, PtCz/PProDOT-Me_2_, and PtCz/PProDOT-Et_2_ ECDs were 24.0, 36.0, and 28.0% at the 3rd cycle, respectively. The Δ*T* of PtCz/PProDOT-Me_2_ and PtCz/PProDOT-Et_2_ ECDs were larger than that of PtCz/PEDOT, indicating PProDOT derivatives facilitated to increase the transmittance disparity when we employed PProDOT derivatives as cathodic layers in ECDs. PtCz/PProDOT-Me_2_ ECD shows the highest Δ*T* among these ECDs, the Δ*T* of PtCz/PProDOT-Me_2_ ECD is higher than those reported for poly(4,4′-di(*N*-carbazolyl)biphenyl)/PEDOT [43], poly(9*H*-carbazol-9-ylpyrene)/PEDOT [37], poly(3,6-bis(2-(3,4-ethylenedioxy)thienyl)-*N*-methylcarbazole)/PEDOT [44], poly(carbazole-*co*-indole-6-carboxylic acid)/PProDOT-Me_2_ [45], poly(4,4′-di(*N*-carbazoyl)biphenyl-*co*-2,2′-bithiophene)/PEDOT [46] and poly(2,5-bis(9-methyl-9*H*-carbazol-3-yl)-1,3,4-oxadiazole)/PEDOT [40] ECDs (Table 6). However, PtCz/PProDOT-Me_2_ ECD shows lower Δ*T* than that reported for poly(4,4′-di(*N*-carbazoyl)biphenyl-*co*-4*H*-cyclopenta[2,1-*b*:3,4-*b*′]dithiophene)/PEDOT ECD [47].

The Δ*T* values of PtCz/PEDOT ECD were 24.0 and 22.2% at the 3rd and 50th cycles, respectively. The Δ*T* of PtCz/PEDOT ECD decreases 1.8% from the 3rd to 50th cycles. Under similar conditions, the Δ*T* values of PtCz/PProDOT-Me_2_ and PtCz/PProDOT-Et_2_ ECDs were 36.0 and 28.0% at the 3rd cycle, respectively, and 35.0 and 26.6% at the 50th cycle, respectively. The Δ*T* values of PtCz/PProDOT-Me_2_ and PtCz/PProDOT-Et_2_ ECDs decrease 1.0 and 1.4% from the 3rd to 50th cycles, respectively. This result indicates that the incorporation of PProDOT-Me_2_ and PProDOT-Et_2_ as cathodic polymers gives rise to better Δ*T* stability than that obtained using PEDOT as the cathodic polymer. 

The *η* value is high when *η* of ECDs is larger than 300 cm^2^∙C^−1^. As summarized in Table 3, the *η*_max_ values of PtCz/PEDOT, PtCz/PProDOT-Me_2_, and PtCz/PProDOT-Et_2_ ECDs were calculated as 256.5, 343.4, and 336.8 cm^2^·C^−1^, respectively, the *η*_max_ value of PtCz/PProDOT-Me_2_ and PtCz/PProDOT-Et_2_ ECDs are larger than that of PtCz/PEDOT ECD, indicating that PProDOT derivatives-based cathodic polymer leads to higher *η* than that of PEDOT-based cathodic polymer. As shown in Table 6, PtCz/PProDOT-Me_2_ ECD shows higher *η*_max_ than those reported for poly(9*H*-carbazol-9-ylpyrene)/PEDOT [37], poly(4,4′-di(*N*-carbazoyl)biphenyl-*co*-4*H*-cyclopenta[2,1-*b*:3,4-*b*′]dithiophene)/PEDOT [47], and poly(4,4′-di(*N*-carbazoyl)biphenyl-*co*-2,2′-bithiophene)/PEDOT [46] ECDs. However, PtCz/PProDOT-Me_2_ ECD shows lower *η*_max_ than that reported for poly(carbazole-*co*-indole-6-carboxylic acid)/PProDOT-Me_2_ ECD [45].

### 3.6. Open Circuit Memory of ECDs

**Figure 9:**
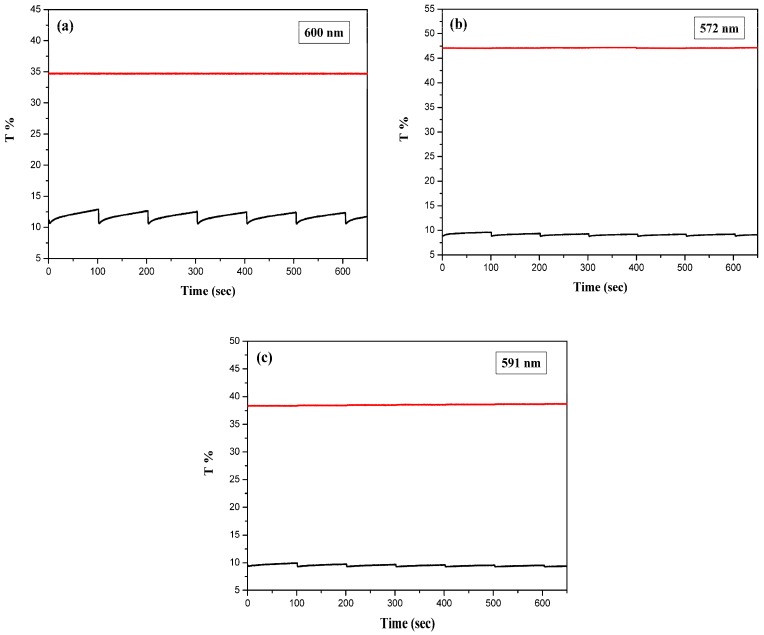
Open circuit stability of (**a**) PtCz/PEDOT (**b**) PtCz/PProDOT-Me_2_, and (**c**) PtCz/PProDOT-Et_2_ ECDs at 0.0 V and 1.8 V. The working electrode is PtCz film-coated ITO glass substrate.

The optical memory of PtCz/PEDOT, PtCz/PProDOT-Me_2_, and PtCz/PProDOT-Et_2_ ECDs were monitored at 600, 572, and 591 nm, respectively, as a function of time at 0.0 and 1.8 V by applying the potential for 1 s at each 100 s time interval. As shown in Figure 9a–c, three ECDs show almost no change of transmittance in the bleached state, i.e., a durable memory effect. The transmittances of three ECDs in the colored state are less stable than in the bleached state, but the transmittance loss is less than 3%. Both the bleached and colored states were highly stable, and the ECDs kept their color without loss, demonstrating PtCz/PEDOT, PtCz/PProDOT-Me_2_, and PtCz/PProDOT-Et_2_ ECDs reveal satisfied open circuit memory.

### 3.7. Long-Term Stability of ECDs

**Figure 10:**
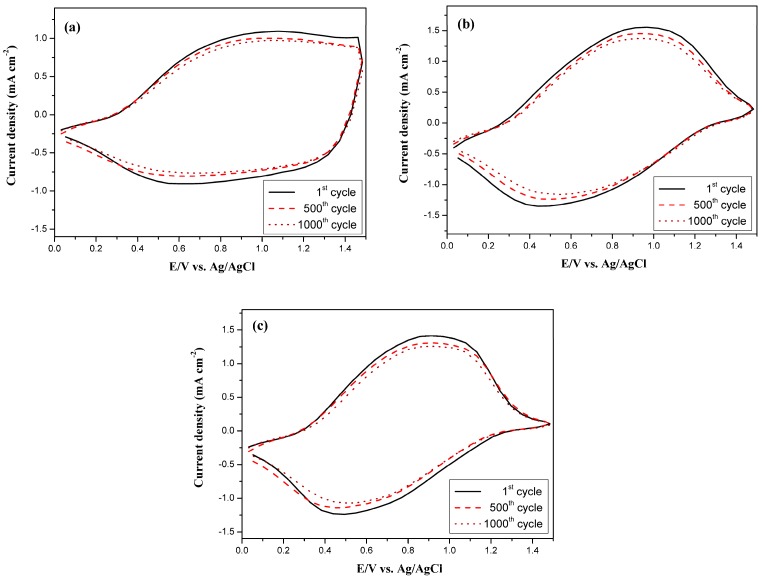
Cyclic voltammograms of (**a**) PtCz/PEDOT; (**b**) PtCz/PProDOT-Me_2_; and (**c**) PtCz/PProDOT-Et_2_ ECDs as a function of repeated with a scan rate of 500 mV∙s^−1^ between 1 and 1000 cycles. The working electrode is PtCz film-coated ITO glass substrate.

The stability of long-term switching between redox states is important for ECDs’ applications [48,49]. The long-term switching ability between redox states of PtCz/PEDOT, PtCz/PProDOT-Me_2_, and PtCz/PProDOT-Et_2_ ECDs were examined using CV at potentials between 0.0 and 1.5 V with a scan rate of 500 mV·s^−^^1^ (Figure 10). From the observation of switching between bleaching and coloring states of the ECDs, 93%, 92%, and 93% of their electrical activities are retained after 500 cycles for PtCz/PEDOT, PtCz/PProDOT-Me_2_, and PtCz/PProDOT-Et_2_ ECDs, respectively, and 87%, 87%, and 87% of their electrical activities are retained after 1000 cycles for PtCz/PEDOT, PtCz/PProDOT-Me_2_, and PtCz/PProDOT-Et_2_ ECDs, respectively, the electrical activities of PtCz/PEDOT, PtCz/PProDOT-Me_2_, and PtCz/PProDOT-Et_2_ ECDs at 500th cycle are larger than those reported for P(BTN-*co*-BT)/PEDOT ECD (stability = 79% at 500th cycle) [50] and PBTBE/PEDOT ECD (stability = 80.2% at 500th cycle) [51], indicating they are good candidates for electrochromic applications.

## 4. Conclusions 

A carbazole-based monomer (tCz) was synthesized, and its corresponding homopolymer (PtCz) was prepared using electrochemical polymerization. The electrochemical processes of PtCz film are reversible, and the PtCz film shows four color variations (light yellow, earthy yellow, grey, and dark grey) from an undoped state to a doped state. Three ECDs based on PtCz as anodic polymer and PEDOT, PProDOT-Me_2_, and PProDOT-Et_2_ as the cathodic polymers were constructed, and the spectroelectrochemical properties of ECDs were characterized. The colors of constructed PtCz/PProDOT-Me_2_ ECD switched from yellowish-grey, light grey, purple, and dark blue upon the application of potential between −0.8 and +1.5 V. Electrochromic switching studies showed that the Δ*T*_max_ values of PtCz/PEDOT, PtCz/PProDOT-Me_2_, and PtCz/PProDOT-Et_2_ ECDs were 24.0%, 36.0%, and 28.0%, respectively, and the *η*_max_ values of PtCz/PEDOT, PtCz/PProDOT-Me_2_, and PtCz/PProDOT-Et_2_ ECDs were calculated as 256.5, 343.4, and 336.8 cm^2^·C^−1^, respectively. Moreover, PtCz/PEDOT, PtCz/PProDOT-Me_2_, and PtCz/PProDOT-Et_2_ ECDs reveal satisfied open circuit memory and long-term switching ability between redox states. The results show that the PtCz film is a potential anodic material for electrochromic applications in rear-view mirrors and motorcycle helmet-visors.

## Figures and Tables

**Table 1:** Feed species of cathodic polymer electrodes (a)–(c).

Electrodes	Cathodic polymer	Feed species	Deposition amount of cathode
(a)	PEDOT	8 mM EDOT	50 mC
(b)	PProDOT-Me_2_	10 mM ProDOT-Me_2_	50 mC
(c)	PProDOT-Et_2_	17 mM ProDOT-Et_2_	50 mC

**Table 2:** Colorimetric values (L*, a*, and b*), CIE chromaticity values (*x*, *y*), and CIE chromaticity diagrams of the PtCz and PProDOT-Me_2_ at various applied potentials.

Electrodes	Potential (V)	L*	a*	b*	*x*	*y*	Diagrams
PtCz	0.0	73.95	5.59	15.85	0.4770	0.4159	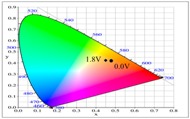
	1.0	74.84	6.13	24.13	0.4864	0.4226	
	1.3	73.46	3.02	21.02	0.4778	0.4248	
	1.5	68.67	−1.78	11.72	0.4588	0.4239	
	1.7	64.66	−3.54	6.65	0.4490	0.4215	
	1.8	61.15	−3.02	5.17	0.4482	0.4193	
PProDOT-Me_2_	−1.5	49.73	6.28	−46.01	0.3710	0.3162	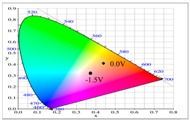
	−1.0	60.60	5.54	−31.57	0.4078	0.6232	
	−0.8	78.43	3.60	−10.50	0.4411	0.3911	
	−0.4	88.43	−2.94	−5.89	0.4362	0.4050	
	−0.2	88.98	−2.68	−5.85	0.4368	0.4048	
	0.0	89.37	−2.56	−5.18	0.4378	0.4053	

**Table 3:** Optical and electrochemical properties investigated at the selected applied wavelength for PtCz film and ECDs.

PtCz film and ECDs	N	*T*_ox_ (%)	*T*_red_ (%)	Δ*T* (%)	ΔOD	*η* (cm^2^∙C^−1^)	*τ*_c_/s	*τ*_b_/s
PtCz (760 nm) ^a^	3	33.0	63.5	30.5	−0.28	54.8	5.5	5.0
PtCz/PEDOT	3	10.4	34.4	24.0	−0.52	234.9	0.6	1.0
(600 nm) ^a^	50	10.8	33.0	22.2	−0.49	256.5	0.2	0.2
Ptz/PProDOT-Me_2_	3	11.6	47.6	36.0	−0.61	248.4	0.2	0.6
(572 nm) ^a^	50	12.0	47.0	35.0	−0.59	343.4	0.2	0.2
PtCz/PProDOT-Et_2_	3	9.3	37.3	28.0	−0.60	336.8	0.4	0.3
(591 nm) ^a^	50	9.8	36.4	26.6	−0.57	330.7	0.1	0.1

^a^ The selected applied wavelength for PtCz film and ECDs.

**Table 4:** Optical contrasts and coloration efficiencies of carbazole-based polymer films.

Carbazole-based polymer films	Δ*T*_max_ (%)	*η* (cm^2^∙C^−1^)	Ref.
poly(9*H*-carbazol-9-ylpyrene)	29 (460 nm)	---	[37]
poly(1,3-bis(carbazol-9-yl)benzene)	18.6 (1050 nm)	180.3	[38]
poly(ethyl-4-(3,6-di(thiophen-2-yl)-9*H*-carbazole-9-yl)-benzoate)	36 (1100 nm)	---	[39]
poly(2,5-bis(9-methyl-9*H*-carbazol-3-yl)-1,3,4-oxadiazole)	75 (660 nm)	---	[40]
poly(3,6-di(carbazol-9-yl)-*N*-(4-nitrophenyl)-carbazole)	52 (710 nm)	35	[29]
poly(4,4′-bis(*N*-carbazolyl)-1,1′-biphenyl)	44.1 (800 nm)	98	[41]
PtCz	30.5 (760 nm)	54.8	This work

**Table 5:** Electrochromic photographs, colorimetric values (L*, a*, and b*), CIE chromaticity values (*x*, *y*), and CIE chromaticity diagram of the PtCz/PProDOT-Me_2_ ECD at various applied potentials.

ECD	Potential (V)	Photographs	L*	a*	b*	*x*	*y*	Diagram
PtCz/PProDOT-Me_2_	−0.8	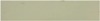	85.06	−1.40	26.92	0.472	0.434	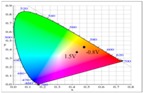
	0.0	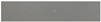	80.27	−0.28	19.04	0.468	0.427	
	0.8	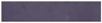	61.82	2.72	−8.38	0.441	0.392	
	1.2	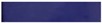	56.30	4.46	−17.66	0.428	0.374	
	1.5	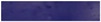	54.18	5.26	−21.30	0.423	0.366	

**Table 6:** Optical contrast and coloration efficiencies of ECDs.

ECD configuration	Δ*T*_max_ (%)	*η*_max_ (cm^2^∙C^−1^)	Ref.
poly(4,4′-di(*N*-carbazolyl)biphenyl)/PEDOT	19 (550 nm)	---	[43]
poly(9*H*-carbazol-9-ylpyrene)/PEDOT	23 (623 nm)	290	[37]
poly(3,6-bis(2-(3,4-ethylenedioxy)thienyl)-*N*-methylcarbazole)/PEDOT	ca. 30	---	[44]
poly(carbazole-*co*-indole-6-carboxylic acid)/PProDOT-Me_2_	32 (575 nm)	372.7	[45]
poly(4,4′-di(*N*-carbazoyl)biphenyl-*co*-2,2′-bithiophene)/PEDOT	28.6 (700 nm)	234	[46]
poly(4,4′-di(*N*-carbazoyl)biphenyl-*co*-4*H*-cyclopenta[2,1-*b*:3,4-*b*′]dithiophene)/PEDOT	39.8 (628 nm)	319.98	[47]
poly(2,5-bis(9-methyl-9*H*-carbazol-3-yl)-1,3,4-oxadiazole)/PEDOT	35 (620 nm)	---	[40]
PtCz/PProDOT-Me_2_	36 (572 nm)	343.4	This work
